# Hybrid Composite Material Reinforced with Carbon Nanolaminates for Gradient Stiffness: Preparation and Characterization

**DOI:** 10.3390/polym13224043

**Published:** 2021-11-22

**Authors:** Alvaro Rodríguez-Ortiz, Isabel Muriel-Plaza, Cristina Alía-García, Paz Pinilla-Cea, Juan C. Suárez-Bermejo

**Affiliations:** Structural Materials Research Center, Universidad Politécnica de Madrid, 28040 Madrid, Spain; alvaro.rodriguez@upm.es (A.R.-O.); isabel.muriel.plaza@gmail.com (I.M.-P.); cristina.alia@upm.es (C.A.-G.); paz.pinilla@upm.es (P.P.-C.)

**Keywords:** hybrid, composite, carbon nanolaminate, gradient properties, stiffness

## Abstract

Currently, the procurement of lightweight, tough, and impact resistant materials is garnering significant industrial interest. New hybrid materials can be developed on the basis of the numerous naturally found materials with gradient properties found in nature. However, previous studies on granular materials demonstrate the possibility of capturing the energy generated by an impact within the material itself, thus deconstructing the initial impulse into a series of weaker impulses, dissipating the energy through various mechanisms, and gradually releasing undissipated energy. This work focuses on two production methods: spin coating for creating a granular material with composition and property gradients (an acrylonitrile–butadiene–styrene (ABS) polymer matrix reinforced by carbon nanolaminates at 0.10%, 0.25%, and 0.50%) and 3D printing for generating viscoelastic layers. The aim of this research was to obtain a hybrid material from which better behaviour against shocks and impacts and increased energy dissipation capacity could be expected when the granular material and viscoelastic layers were combined. Nondestructive tests were employed for the morphological characterization of the nanoreinforcement and testing reinforcement homogeneity within the matrix. Furthermore, the Voronoï tessellation method was used as a mathematical method to supplement the results. Finally, mechanical compression tests were performed to reveal additional mechanical properties of the material that had not been specified by the manufacturer of the 3D printing filaments.

## 1. Introduction

Because multiple combinations may arise from two materials of different natures (organic and inorganic), the field of nanoscience has prioritised the design and production of new hybrid structural materials that can exceed their constituents in terms of properties [1]. In fact, increasingly lighter and stronger materials with high-energy dissipation and damage tolerance capacities are becoming essential for several industrial applications. Without weight restrictions, several materials have been shown to be highly effective in dissipating the mechanical energy received from an impact. However, whenever the use of these materials is not feasible due to density or thickness limitations, new structural hybrid materials without these limitations are required. Furthermore, some current theoretical models focus on capturing energy within a granular medium. Subsequently, the confined energy is slowly and gradually dissipated; this is achievable if the particles are conveniently distributed in layers of different particle sizes, in layers of different thicknesses, or in evenly separated layers. In this context, the final goal of this study is to propose combined mechanisms that will represent a great step forward from the energy dissipation mechanisms known so far, as it may provide significant improvements in existing materials [2,3] for protecting against low energy impacts.

However, materials with a continuous impedance gradient have been designed in the last few decades [4] for different purposes, and some of them are graded composites based specifically on polymers [5]. These materials allow the impact energy to dissipate through multiple reflections to prevent material failure. In these cases, as has already been demonstrated in the literature [6], the dissipation of energy increases with increasing impedance difference between layers. However, failure at the interfaces may be caused primarily by high concentrations of stress and changes in the direction of wave propagation. Therefore, this study focuses on creating a material without interfaces that exhibits continuous impedance variations and prevents internal reflections.

Furthermore, biomimicry and bioinspiration are useful for the development of new hybrid materials because many tenacious biological materials have remarkable energy absorption capacities because of the reinforcement mechanisms operating at different spatial scales. For example, the prehistoric *Polypterus senegalus* fish protects itself using its natural scale armour, composed of four layers of organic–inorganic nanocomposites with gradient properties [7].

This work seeks to combine the aforementioned strategies to develop a tough, lightweight hybrid material (with high-energy dissipation capacities and low density) that may be used as a passive protection layer in vehicles. With this goal in mind, this work proposes a new material with a hierarchical granular structure that combines different energy dissipation mechanisms that have already proved to be effective in previous investigations. Because of their structure, each mechanism will be effective for a specific range of energies and for temporarily confining energy to be gradually dissipated at a later time.

As the first step in this study, a granular polymer matrix material was produced as a filament for 3D printing at a given percentage. For these purposes, nondestructive tests were conducted to characterize the reinforcement and composite material. The added reinforcement layer comprised carbon nanolaminates, commonly known as graphene nanoplatelets, because they offer uniform isotropic reinforcement loads added, even when they are randomly oriented (as long as the aspect ratio of the particles is sufficiently high) [8]. In addition, these loads have low density, high resistance, good thermal and electrical conductivity, low thermal expansion coefficient, and high thermal stability [9,10,11].

Numerous researchers have considered adding different amounts of this reinforcement to a polymer matrix to subsequently evaluate the behaviour of the material with respect to some of its main mechanical properties. In this sense, the authors have confirmed that adding graphene nanoplatelets to a thermostable matrix induces greater rigidity; however, other mechanical properties, such as resistance and elongation, decrease due to the weak bonding of the matrix-reinforcement interface [12]. Other authors have demonstrated the ability of graphene nanoplatelets in a laminate to absorb and dissipate impact energy at low speeds, as well as their contribution to improving flexural strength and fracture toughness [13,14]. However, these studies assert that the number of graphene nanoplatelets that can be added to a matrix is limited because any excess could detrimentally affect its properties and multiply its processing issues.

Second, 3D printing techniques can be used to create viscoelastic layers. These techniques are widely applied in several fields and are commonly characterized by their versatility and process automation. Furthermore, 3D printing can be used to bring a previously modelled digital design to life in a short period of time at an affordable cost [15,16,17,18]. In fact, this technique offers a wide array of possibilities in this study because it is able to generate layers using different materials and create various configurations, even with complex geometries.

In addition to the viscoelastic layers manufactured by the 3D printer, the spin coating technique is used to produce thin layers and create a hierarchical structure by combining several such layers (with different loading percentages added to the polymer matrix) to grade the composition of the granular material and, in turn, obtain a gradual variation of its mechanical properties. The effect of the shape and size of the particles plays a fundamental role in the convenient distribution of the reinforcement in the polymer [19]. Spin coating (coating by centrifugal force) of a polymer solution on a substrate is a convenient method for manufacturing thin and ultrathin films, with ultrathin films defined as films with thickness <1 µm. The film thickness is adjusted by changing the concentration and viscosity of the polymer solution, as well as the spinning speed [20,21,22].

As discussed above, the introduction of the configuration achieved by combining the viscoelastic layers obtained through 3D printing and the multiple gradient layers produced through spin coating into a structural material may improve its resistance to shocks and impacts, as well as its capacities for dissipating energy through different mechanisms.

Throughout the development of this study, the correlation between virtual and experimental testing is considered critical. In our experimental phase, nondestructive techniques, such as SEM and microcomputed tomography (micro-CT), will be used for morphological characterization and assessing the homogeneity of the reinforcement distribution, respectively. From these data, a new mathematical code was created to obtain new results based on Voronoï diagrams. From the results of the tests conducted at different scales, we will be able to model the macroscopic behaviour of the material on the basis of the molecular and microscopic data previously obtained. Therefore, the combination of virtual and experimental tests optimizes the composition, distribution, and design properties, while minimizing development times and costs.

## 2. Materials and Methods

**Figure 1:**
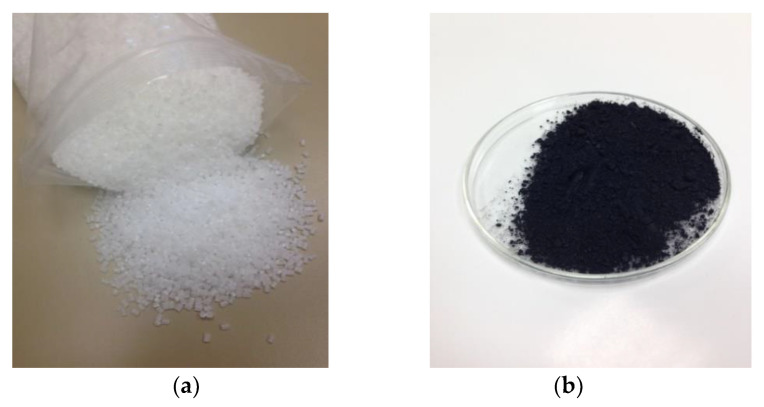
Materials used in the hybrid layers: (**a**) ABS pellets and (**b**) carbon nanolaminates.

The materials used for 3D printing the viscoelastic layers, which will be placed inside the thin layers of the composition gradient built by the spin coating technique, are ABS (blue) and thermoplastic polyurethane TPU 95A (white), both provided by the Ultimaker company from the Netherlands. The MAGNUM 8391 ABS from Trinseo, distributed by Channel Prime Alliance LLC, USA, was used to manufacture the filament and to manufacture the thin layers in the composition gradient. White acrylonitrile butadiene styrene (ABS) pellets were used for the polymer matrix. Furthermore, as additional reinforcement, we used carbon nanolaminates provided by avanGRP AVANZARE Innovación Tecnológica SL, Spain, with a particle size of 2 × 5 μm^2^ and a thickness of less than 10 nm. Carbon nanolaminates are a viable alternative to using carbon nanotubes in nanocomposites because of their excellent mechanical and structural properties (at traction and impact), as well as to their good thermal and electrical conductivity. The extreme hardness of graphene and carbon nanolaminates combined with their malleability and lightness turn these materials into an ideal shielding compound. Figure 1 shows a sample of these two materials.

Furthermore, acetone, rather than chloroform, which has been used in other reference studies [23,24], was used as a solvent to mix these two materials to obtain thin layers.

For the manufacturing of the carbon-nanostructure reinforced film, we produced an ABS filament reinforced with 0.25% carbon nanostructures (CNS) as well as other compositions. To obtain 100 g of the total mixture, we used 99,750 g of ABS and 0.250 g of CNS. The components were mixed mechanically, and the resulting material was subsequently smelted for extrusion. Then the filament was produced through the FilaFab Pro 350 EX extruder model at an extrusion temperature of 237.5 ° C and using a 1.2 ± 0.045-mm nozzle. The final diameter of the resulting filament was approximately 1.25 mm. In addition, a spooler was manufactured to facilitate spooling and ensure the consistency of the filament.

**Figure 2:**
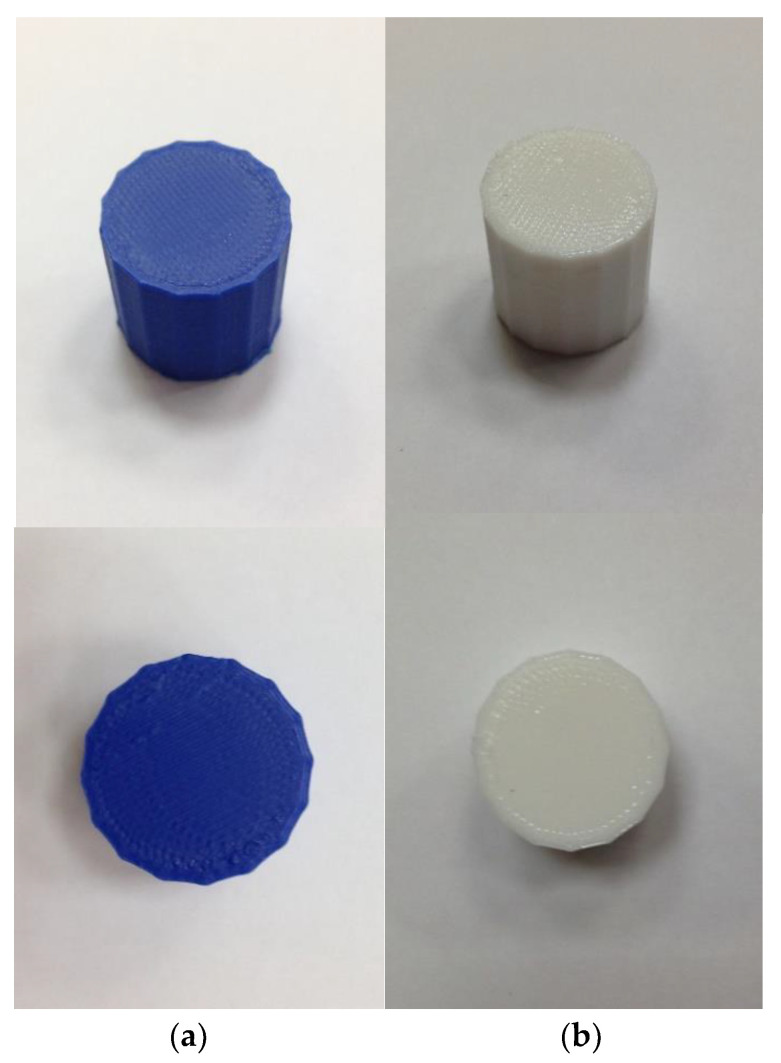
Samples printed on the 3D printer for compression testing: (**a**) ABS and (**b**) TPU.

To supplement the mechanical properties provided by the manufacturer in the data sheet, five hexadecagon prism samples of each material—ABS (Acrylonitrile butadiene styrene) and TPU 95A (thermoplastic polyurethane)—were assembled with an Ultimaker S3 3D printer with heated buil plate and dual extrusion. The nozzle temperature was 230 °C. These samples were used for compression tests to obtain the modulus of elasticity and the elastic limit. Figure 2 shows the samples generated from both materials before testing.

**Figure 3:**
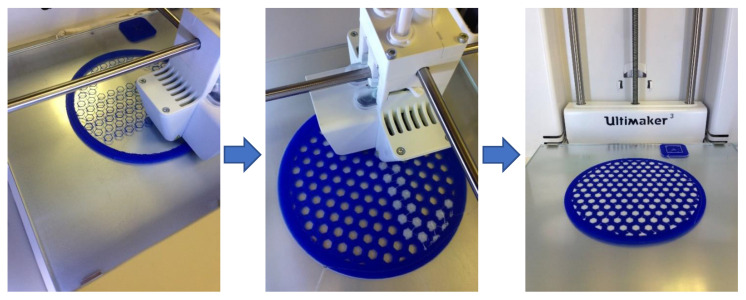
Printing process for a viscoelastic layer combining ABS and TPU in the double head 3D printer.

The 3D printer produced viscoelastic layers with a diameter of 120 mm and a thickness of under 1.5 mm. The internal geometry of the ABS (blue) layers mimics a honeycomb, with its hexagonal cells filled inside with an elastomeric material. In this case, this material is TPU 95A (white). All parts made with the 3D printer had been designed using the CATIA computer-assisted design software. In addition, the Cura freeware was used to process design files and print in 3D. Figure 3 displays the different stages of the 3D printing process.

**Figure 4:**
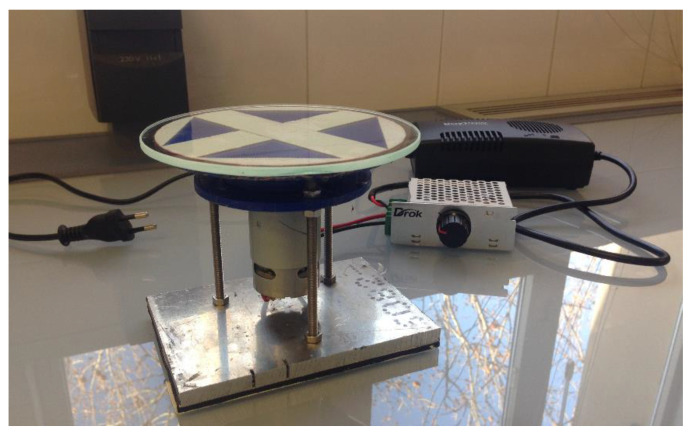
Spin-coating equipment for the production of thin films (without protective casing).

To use this technique to obtain thin films (thickness < 1 mm), the corresponding equipment shown in Figure 4 had to be manufactured. This included a glass disk attached to a DC electrical motor and a protective casing to prevent the polymer from spreading out of the support. This image shows the current transformer and inverter required to start the equipment and control its spin speed; however, the image does not show the methacrylate tube used as protection during the equipment operation.

Some pieces of this equipment have been redesigned to solve the weakness issues presented by parts that were initially 3D printed; thus, the equipment performance was improved. The equipment can achieve speeds of up to 5000 rpm. However, different spinning speeds were measured using an optical tachometer with a microprocessor (PCE-T 260) and marked on the inverter. In all cases, the nanostructure volume used for thin layer production was less than 1% (to provide reinforcement that does not compromise other properties). The different percentages of reinforcement added to the polymeric matrix to create a composition gradient and a gradual variation of the mechanical properties of the material by forming thin layers are 0.10%, 0.25%, and 0.50% of the nominal content of the material by weight. Table 1 displays the amounts used.

The quantities were weighed using a PJ360 precision balance with an accuracy of 0.001 g, and the amount of acetone added to each mixture is measured using a laboratory graduated test pipe.

- Mixture A: 50 g ABS + 100 mL of acetone for dissolution. Manual mixing is combined with magnetic stirring (at an approximate speed of 500 rpm) until the mixture is free of lumps.
- Mixture B: Corresponding amount of CNS according to concentration + 50 mL of acetone. Place mixture B in an ultrasonic bath for 15 min to disperse the CNS.
- Mixture C = Mixture A + Mixture B: Mixture B was poured into the Mixture A container while being agitated by the magnetic stirrer to favor the mixture.

After 48 to 72 h, at an approximate working temperature of 17 °C, the mixture reached the appropriate viscosity to allow the generation of thin layers using the spin coating equipment that was specifically manufactured in the laboratory workshop for this study. To start creating a layer, the equipment was started at a speed of 2800 rpm. Using a syringe, 4 mL of the mixture with a lower concentration (0.10% CNS) was poured into the stirrer, keeping the syringe perpendicular to the turntable, and discharging the material in less than a second. This is the most essential step of the entire process because it is responsible for maintaining a homogeneous thickness. After the solvent has evaporated (after approximately five minutes), the next layer, which had a concentration of 0.25% CNS, was placed. Then, we repeated the same process for the last layer at a concentration of 0.50% CNS. Quantities, times, and speeds were also replicated. Finally, we had to wait at least 30 min to detach the multilayer from the turntable to prevent it from deforming.

**Figure 5:**
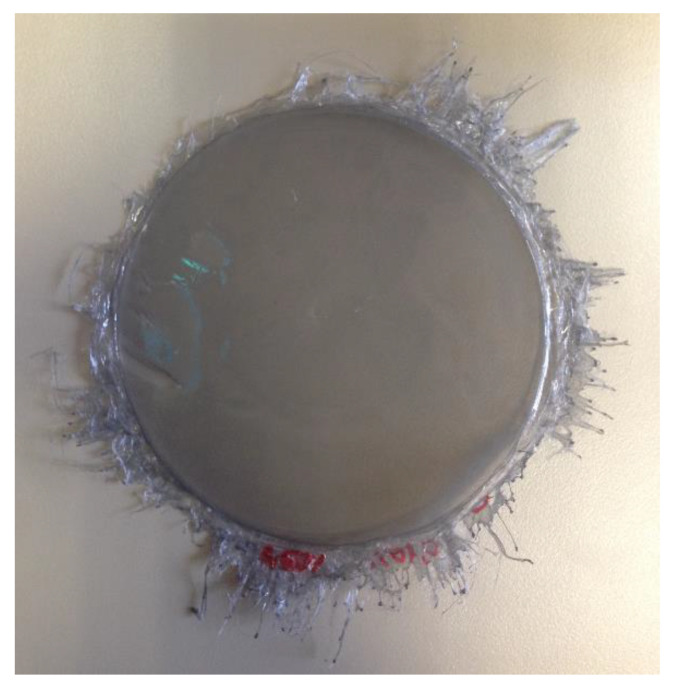
Multilayer film created by spin coating, composed of 3 reinforced ABS layers reinforced with 0.10%, 0.25%, and 0.50% CNS, respectively.

Using this technique, the cast layers are perfectly bound to each other. The thickness of this multilayered film is 0.09 to 0.12 mm, and the thickness of each single layer is 0.03 to 0.04 mm. Figure 5 shows the results of a multilayer gradient. The threads that can be observed around the layers are surplus material that is detached during the spinning used to create the layers.

**Figure 6:**
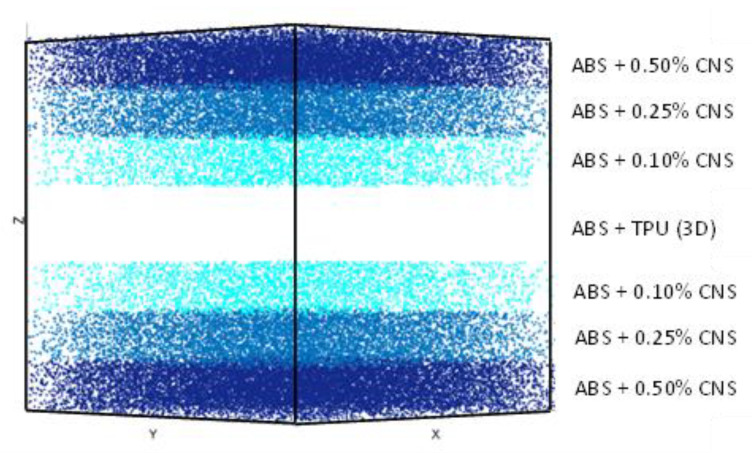
Layer scheme combining the obtained spin-coating multilayers with the 3D printed viscoelastic layers.

Figure 6 denotes the preliminary design layout for the combined viscoelastic layers (3D printing plus spin coating) viscoelastic layers. The multiple layers created through spin coating are arranged on top and under the plates manufactured by the 3D printer from highest to lowest concentration (from highest to lowest stiffness). The incoming impacts are decomposed into numerous smaller pulses that are dissipated by the internal movements of the CNS. The gradient composition ensures that impact waves are reflected at interfaces, and consequently, a higher amount of energy is absorbed by the protective shielding layers. This mechanism has been proposed by some authors [6,7], but so far there are few examples of actual materials being designed and manufactured according to these principles. Once introduced within a structural material, this configuration allows the TPU placed as the central layer (thus creating a symmetry plane) to be compressed without any freedom to expand, constrained in such a way that the energy produced by the impact is returned after rebound of the impactor. The thickness of these viscoelastic layers plus the thickness of the gradient layers is less than 2 mm.

**Figure 7:**
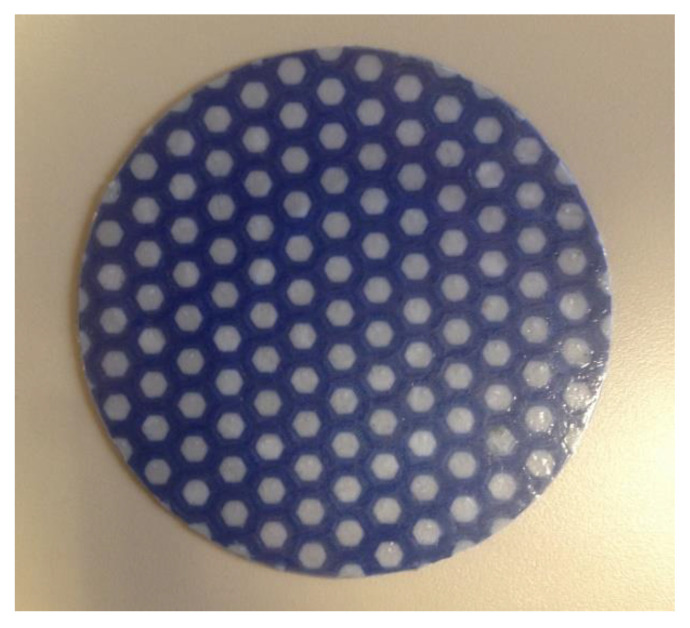
Final viscoelastic layers combining 3D printing and spin coating layers.

For bonding the multiple gradient layers to the viscoelastic layer, the configuration shown in the image was followed. Once the different layers were laid, they were placed between two heating plates at 100 °C for 120 min, and a uniform pressure of approximately 500 Pa was applied to facilitate the compaction of the layers. Then, each layer combination was allowed to cool slowly under the same pressure to prevent any deformations. After 24 h, the sample was removed from the heating plates. Figure 7 shows the results obtained.

Nondestructive SEM and microcomputed tomography techniques were used to morphologically characterize the reinforcement before adding it to the matrix and to verify its distribution homogeneity in the matrix during filament production. Furthermore, destructive compression tests were conducted to obtain the modulus of elasticity and elastic limit of the materials used in the 3D printer and to supplement the technical information provided by the manufacturer. For characterization using SEM, two samples with the same concentration were prepared by mixing CNS with methanol but using different preparation methods. In the first sample, carbon and methanol were mixed manually before a drop of the mixture was poured into the sample holder. In the second sample, after manual mixing, the sample was placed in an ultrasonic bath for 15 min before a drop of the mixture was deposited in the sample holder. In addition, both samples were metallized to make them conductive for assessment by SEM. The equipment used for this analysis was the 6400 JSM scanning electron microscope from JEO. The micro-CT equipment used for this analysis is the SkyScan 1272 from Bruker. For the purposes of this analysis, the reinforcement amount in a filament specimen was 0.25% CNS. This amount of reinforcement added was verified based on the difference in densities because the density of the CNS is less than 0.2 g/cm^3^ and that of the ABS is 1.05 g/cm^3^ according to the technical data sheets provided by the manufacturers.

Compression testing was performed using 0T Servosis ME/401/10 dynamic test equipment. Testing was carried out at a speed of 3 mm/min until the samples were plastically deformed (only in the case of ABS since the TPU samples have not been plastically deformed). The displacement was recorded using a 3541 clip-on-gages extensometer from Epsilon Technology Corp. Furthermore, for the purposes of these tests, 5 hexadecagon prism samples were manufactured using the 3D printer.

The large amount of data obtained through the microcomputed tomography was analysed using a mathematical code suitable to verify the homogeneity of the granular material distribution and obtain other useful information for future virtual models. Therefore, the Voronoï regions for each particle have been calculated by adapting the MATLAB code “Polytope bounded Voronoï in 2D and 3D”, shared through MathWorks [25]. This software provides numerous functions and calculates the Voronoï diagram for a finite set of points limited by an arbitrary polytope from the Delaunay triangulation. Nevertheless, its code has been modified to input the mass centre values of the reinforcement particles from the assessed sample, and cubic volume limits have been defined for the particles. Despite the complexity of the program, the concept of Voronoï diagrams is rather simple. Given a set of points, each point is linked to other neighbouring points. If we can map the equidistant border from the first point or seed to the remaining points, we can determine a region in the spatial plane known as the Voronoï region or cell. In fact, the Voronoï diagram is based on Delaunay triangulation, one of the most widely used mathematical algorithms.

From the results obtained in the calculation of the Voronoï regions, the distance between particles is determined to assess whether the particles are uniformly distributed. The distance between particles was measured using Equation (1):

(1) $$d=\sqrt{{\left(x_{2}-x_{1}\right)}^{2}+{\left(y_{2}-y_{1}\right)}^{2}+{\left(z_{2}-z_{1}\right)}^{2}},$$

The differences between brackets represent the distance between two points in all three coordinates of space.

## 3. Results

**Figure 8:**
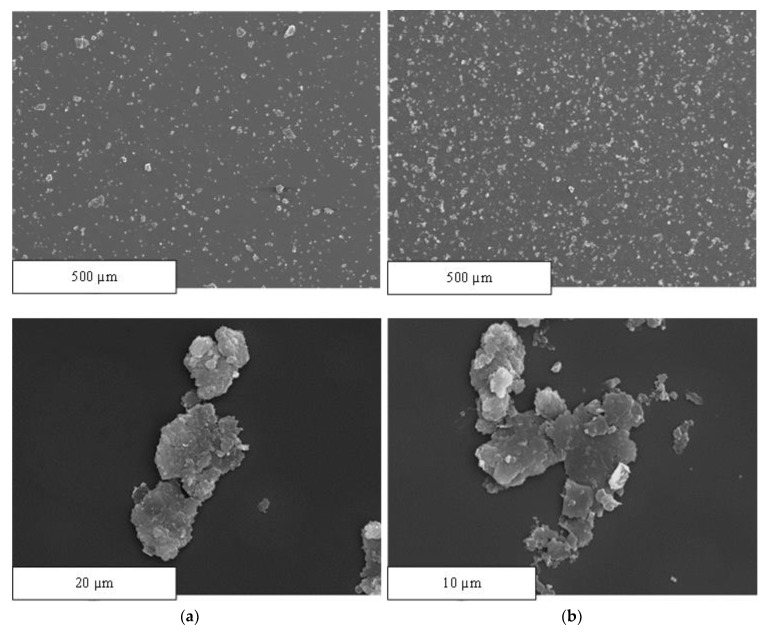
SEM images of carbon nanostructure samples: (**a**) sample prepared without ultrasound (×100 and ×2500), (**b**) sample prepared with an ultrasonic bath after manual mixing (×100 and ×4500).

In Figure 8, after comparing images a and c, we may observe that placing the samples in an ultrasonic bath favours particle dispersion. These images were taken at the same magnifications (×100) and on the same scale (500 μm). However, there is a noticeable difference in the size and shape of the particles, as evidenced in Figure 8a,b. The theoretical dimensions of the CNS are 2 × 5 μm^2^ and less than 10 nm thick, as discussed above. However, we have not been able to find a single nanostructure with those dimensions. In fact, these nanometric particles tend to group together and form clusters where some particles are on top of the others. However, as a consequence of the preparation of the sample using ultrasonic stirring, the particles tend to disperse and disaggregate.

Furthermore, we found inconsistencies in the nomenclature of this reinforcement material. This study refers to CNSs and not graphene nanoplatelets, as specified by the manufacturer and as demonstrated by this analysis. However, numerous authors refer to graphene nanoplatelets as carbon allotropes in a planar sheet of one atom thick, formed by several stacked graphene layers (ranging from 1 to 10) spaced at a distance greater than that between graphite sheets [26].

**Figure 9:**
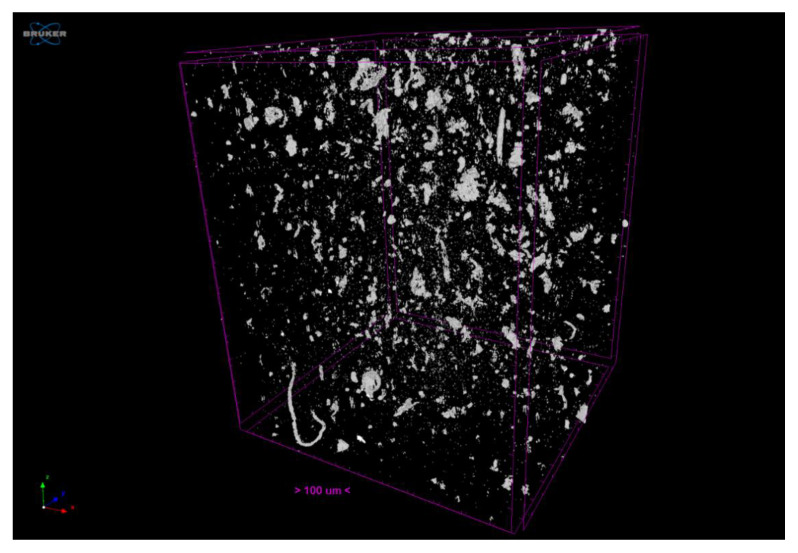
3D reconstruction based on images obtained from micro-CT analysis.

Instrumental SEM and micro-CT techniques are of great importance in characterization of the distribution of fillers in polymer composites [27,28]. Figure 9 shows the volume of the sample evaluated (1200 × 1200 × 1500 μm^3^) by microcomputerized tomography analysis. In this image, the prism edges in fuchsia delimit the analysis sample, while the white particles correspond to the carbon nanostructure clusters. Furthermore, this software-generated image graphically denotes the reinforcement material distribution. In this paper, by identifying the mass centre of each particle, we can analytically determine whether this distribution is homogeneous.

**Figure 10:**
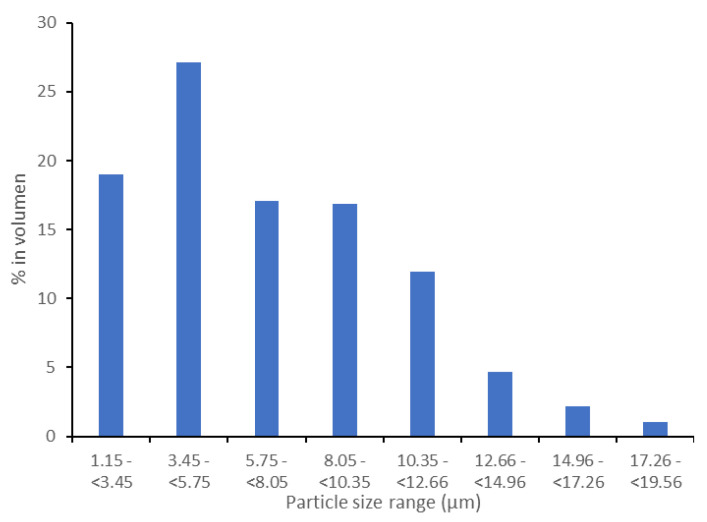
Particle size distribution of the ABS filament reinforced with 0.25% CNS.

According to the analysis, the percentage of ABS polymer matrix is 99.88% (0.12% of CNS). The significant difference between the carbon-nanostructure percentages and the percentages reported in the literature (0.25%) may be due to the manufacturing method. In the filament fabrication process, the material that remains stuck to the walls of the mixing container is lost. In fact, reinforcement material continued to be lost in the extruder hopper even after being mixed (but not yet in the molten state). The decrease in the percentages of reinforcement material may also be due to the analysis being focused on the central area of the sample, thus avoiding the edges. However, material losses in the manufacturing process are inevitable, and an additional percentage must always be added to offset these losses. As a direct result of this test, the particle size distribution is denoted in Figure 10.

**Figure 11:**
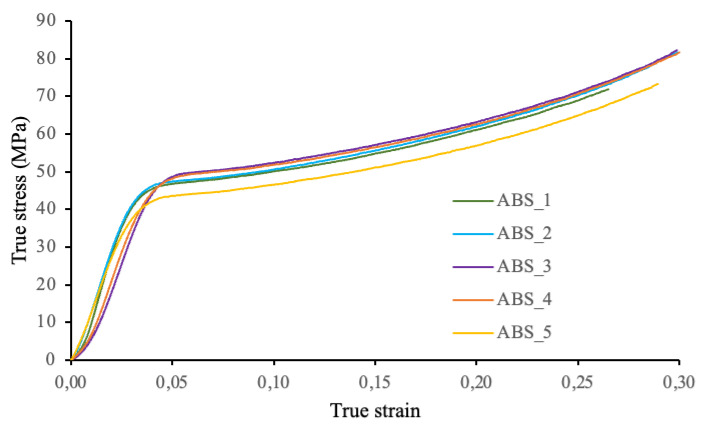
True stress-strain curves for ABS samples.

**Figure 12:**
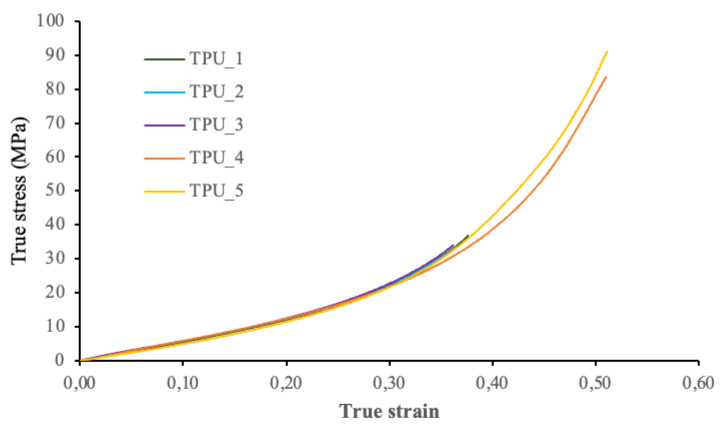
True stress–strain curves for TPU 95A samples.

Figure 11 shows the stress–strain curves for each of the five ABS samples tested, while Figure 12 shows the stress–strain curves for the TPU 95A samples tested.

It should be mentioned that in the TPU_4 and TPU_5 tests, the extensometer was not placed in the samples. Therefore, the values used in the calculations were taken from the crosshead displacement of the testing machine. On the basis of the previous curves, we may observe that these test samples show a greater deformation than the others tested with an extensometer. The results are shown in Table 2.

For ABS samples, a mean modulus of elasticity value of 1546.4 MPa and a mean elastic limit value of 49.3 MPa were reported. The mean modulus of elasticity obtained for the TPU samples is 63.7 MPa. To perform these tests, the ABS_1, ABS_2, and ABS_5 specimens were placed on a support base (the last layer manufactured in 3D printing). The ABS_3 and ABS_4 specimens were supported on the other base (the first manufacturing layer in contact with the 3D printing plate). Regardless of how the test samples are placed (on one prism base or another), the base that touches the printing face during the manufacturing process is always less deformed. This may be due to the type of surface because the base that is in contact with the printing face is significantly smoother.

## 4. Discussion

**Figure 13:**
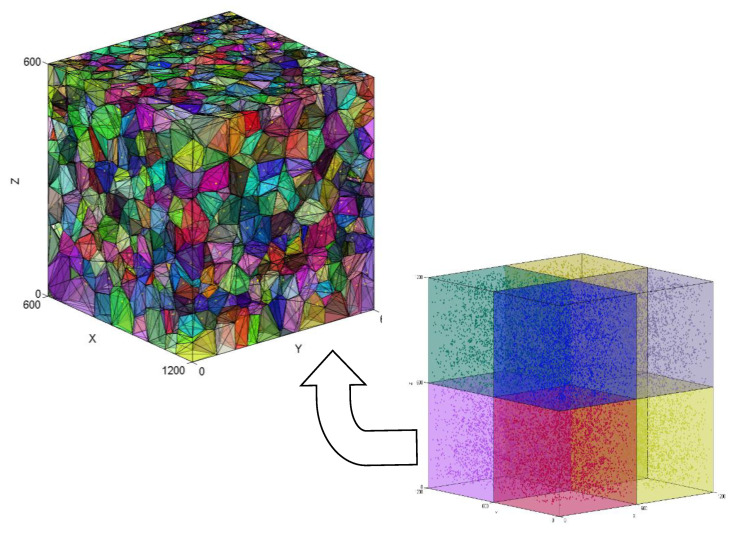
Analysis sample representation divided into 600 × 600 × 600 μm^3^ cells and Voronoï regions for one of these cells.

From the mass centres of each particle found in the analysis survey in the microcomputed tomography assessment, the homogeneity of the reinforcement material can be verified analytically. The volume of filament analysed in this section is a 1200-μm^3^ cube, which is slightly lower than the total volume of the filament sample assessed by micro-CT. Consequently, after knowing the sample volume and the number of particles found in that volume, the average volume for each Voronoï region must be close to a value of 62,852 μm^3^. To avoid calculation errors due to the large number of particles, the total volume of the sample is divided into eight smaller 600 × 600 × 600 μm^3^ cells, as shown in Figure 13. The total volume was also divided into 300 × 300 × 300 μm^3^ cells, but to simplify visualization, these values will not be displayed. To validate the uniformity of the reinforcement material distribution, the volume of the Voronoï regions must remain close to the value calculated previously, regardless of cell size.

Table 3 displays the average volume of the Voronoï regions for each of the cells into which the total volume has been divided, as well as their mean standard deviations and the coefficient of variation. Furthermore, this table shows the number of particles in each cell and the percentage they represent compared to the total volume. From these values, we may observe the following:

- The average number of particles per cell is approximately 3437, implying that for a total of 27,493 particles, each cell represents 12.5% of the total number of particles assessed.
- Numerically, we can verify that the average volume of the Voronoï regions for the particles in each cell is approximately 62,000 μm^3^, which is close enough to the theoretical calculated value (63,000 μm^3^).
- The mean coefficient of variation (V = σ/x) for cells 1, 3, 5, 6, 7, and 8 is less than 1.5%. The lower the coefficient of variation, the lower the heterogeneity of the distribution.
- By comparing the data of each cell, we can safely confirm that the reinforcement material used in this sample is homogeneously distributed because even when the deviation is slightly different and greater for cells 2 and 4, the overall values remain close.

**Figure 14:**
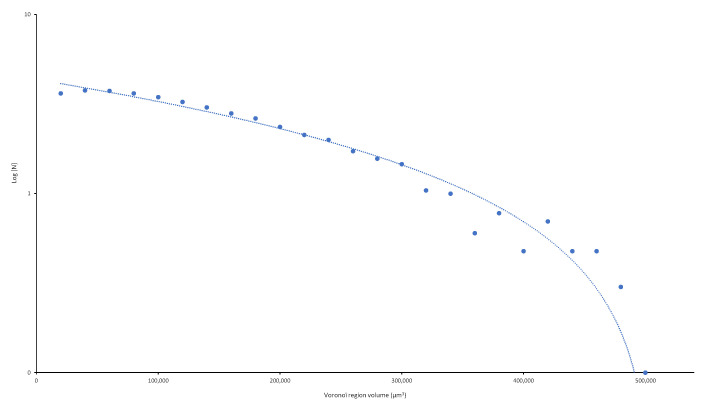
Number of particles (N) for each Voronoï volume range.

Figure 14 shows the number of particles estimated for each Voronoï volume range. From these numerical values, we may observe the following:

- The Voronoï volume for the largest number of particles is within the 20,000–40,000 µm^3^ range.
- The sample areas with a greater number of particles show smaller Voronoï region volumes and, therefore, smaller standard deviations.
- Theoretically, an average Voronoï volume close to 60,000 means that the particles are homogeneously distributed. However, we can safely assert that the Voronoï volumes and the particle distribution exhibit some homogeneity in the 20,000 to 80,000 µm^3^ range since the coefficient of variation is less than 1%.

**Figure 15:**
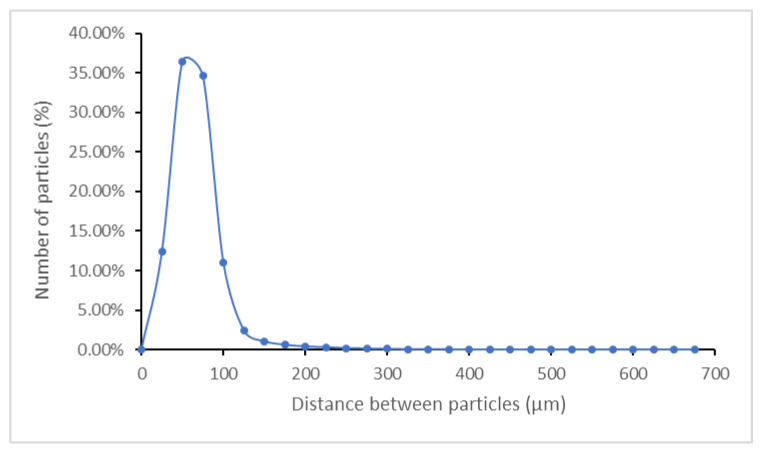
Distance distribution between particles.

Based on the calculation of Voronoï regions and the identification of their neighbouring particles, we can use Equation (1) to determine the distance between particles as an alternative method to check whether the CNS are homogeneously distributed in the ABS matrix. The resulting graph can be observed in Figure 15.

The results reveal that the most common distances at which particles are found are within 0–100 µm. Furthermore, most of the particles are at a distance of 50 µm. Therefore, since most particles (specifically 94.5%) can be found within this range (0–100 µm) and at a calculated average distance of 50.2 µm, the distance between particles remains relatively close; therefore, carbon nanoparticles are homogeneously distributed in the matrix. However, particles of up to 675 µm have occasionally been spotted (<0.001%) during the micro-CT survey of the samples.

The stress–strain curves of the test samples for each material were used to calculate their elastic modulus values by determining the slope of the straight section of the corresponding curve. To calculate the yield stress of each sample, a parallel line was drawn to the straight section of the curve with a deformation of 5%. The point where the parallel line intersects the curve represents the maximum stress supported by the material before permanent deformation is achieved. For thermoplastic polyurethane (TPU), the yield stress was not calculated because the samples were not plastically deformed.

The proper distribution of the particles in the composite material is a key parameter in assessing the performance of the reinforcement. It is also of paramount importance the average distance between particles and the volume of polymer around every of these particles. During impacts events, particles move and clash with their closest neighbours. When the average distance is too large, the interaction among particles is weak and the reinforcement does not work properly. However, when too many particles clutter the polymer, there is not enough matrix to dissipate the shock wave energy, and the shielding effect is less effective. The Voronoï analysis gives valuable information on the actual values of these microstructural parameters in the thin layers with gradient composition. The capacity of these hybrid materials to be effective protective shielding materials has been tested by integrating the layers inside a composite panel and analysing the residual strength under compression after being impacted by a vertical weight drop tester. Dissipated energy as a function of impact energy, damage generated in the GFRP laminate (with and without shielding hybrid viscoelastic layers), and residual strength results have been recently published in this same journal and elsewhere [29,30].

## 5. Conclusions

In this research study on the design, manufacture, and characterization process of a hybrid granular material with gradient composition and stiffness, the following conclusions have been reached:

- The spin-coating technique used to produce thin layers of granular material (ABS reinforced with different percentages of carbon nanostructure) provides accurate results to obtain controlled stiffness layers with thickness below 0.04 mm. Furthermore, these thin layers may be bound with viscoelastic layers generated by 3D printing (a combination of ABS and TPU 95A) with a hexagonal cell pattern. Graded stiffness layers and constrained deformation of the elastomeric material (TPU) by the ABS cell walls are known to be effective energy dissipation mechanisms impacting the plates. This remains to be experimentally confirmed with drop-weight tests that are part of ongoing research.
- Nondestructive tests (specifically SEM and Micro-CT analysis) were performed to morphologically characterize the spatial distribution of the reinforcement material (CNT) and verify the homogeneity obtained by mixing the reinforcement particles with the polymeric matrix using ultrasonic stirring.
- SEM analysis revealed that even when the particles exhibit differences in size and shape, they are grouped together in clusters in which one is placed on top of the other. Therefore, we are not working with pure graphene platelets; therefore, this paper uses the term “CNS”, which stands for carbon nanostructures.
- MicroCT analysis proved to be a useful technique for verifying the homogeneity of the reinforcement distribution of an ABS filament sample reinforced with CNS. The particle size distribution and average volume around every CNS have been determined from the tomographic data provided by the x-ray images.
- Calculation of Voronoï regions (volumes around every CNS) is an effective method to test the reinforcement distribution in the analysis sample. Based on 3D representations and subsequent evaluation of results (performed in 75.7% of source data), the CNS was homogeneously distributed throughout the sample, even when the particles tend to cluster and exhibit slight differences in their size. More than 27,000 individual particles have been statistically analysed.
- On the basis of Voronoï diagrams and finding the distance between particles, the matrix-reinforcement mixture can be asserted as homogeneous. The volumes of the Voronoï regions have been evaluated from 20,000 to 500,000 µm^3^, and an average distance between the CNS of 80 µm has been determined. Actual values for these microstructural parameters in the polymer with gradient composition are of great importance for assessing the role as a shielding protective layer of this hybrid material.
- In compression tests, the modulus of elasticity value obtained for ABS (1546.4 MPa) is similar to the tensile modulus of elasticity reported in the data sheet (1681.5 MPa). For the TPU, as reported in the test results, the tensile modulus of elasticity is 26.0 MPa, while the compression modulus of elasticity is 63.7 MPa. Control of stiffness is of paramount importance for the targeted use of these layers as protective shielding materials for composite panels under impact loading.

## Figures and Tables

**Table 1:** Quantities used to mix different concentrations and create multiple layers.

Mixture A (Pure ABS)	Mixture B (ABS + CNS)	Mixture C
50 gr ABS/100 mL acetone	50 gr ABS + 0.050 gr CNS/50 mL acetone	A + B
	50 gr ABS + 0.125 gr CNS/50 mL acetone	A + B
	50 gr ABS + 0.250 gr CNS/50 mL acetone	A + B

**Table 2:** Elastic modulus and yield stress for ABS and TPU samples.

ABS	Elastic Modulus (MPa)	Yield Stress (MPa)	TPU	Elastic Modulus (MPa)	Yield Stress (MPa)
ABS_1	1852.9	48.7	TPU_1	63.2	-
ABS_2	1615.0	49.2	TPU_2	64.0	-
ABS_3	1354.0	52.0	TPU_3	64.7	-
ABS_4	1488.7	51.0	TPU_4	67.8	-
ABS_5	1421.6	45.4	TPU_5	58.8	-
Average	1546.4	49.3	Average	63.7	-

**Table 3:** Data obtained from the Voronoï regions of each cell into which the total volume was divided.

Cells (Volumen = 600 × 600 × 600 µm^3^)	Voronoï Region Average Volume (µm^3^)	Standard Deviation	Variability Percentage (%)	Number of Particles	% of Particles in the Total Volume
Cell 1	60,279	551	0.915	3582	13.029
Cell 2	59,969	1713	2.857	3603	13.105
Cell 3	66,682	696	1.043	2828	10.286
Cell 4	70,622	1525	2.160	3056	11.116
Cell 5	57,383	658	1.146	3766	13.698
Cell 6	57,868	583	1.007	3731	13.571
Cell 7	61,346	560	0.912	3520	12.803
Cell 8	63,399	575	0.906	3407	12.392
Average	62,194	858	-	-	-
Total	-	-	-	27,493	100.000

## Data Availability

Not applicable.
